# MiR-181a contributes to bufalin-induced apoptosis in PC-3 prostate cancer cells

**DOI:** 10.1186/1472-6882-13-325

**Published:** 2013-11-23

**Authors:** Xiao-feng Zhai, Fan-fu Fang, Qun Liu, Yong-bin Meng, Yu-yu Guo, Zhe Chen

**Affiliations:** 1 Department of Integrative Oncology, Changhai Hospital of Traditional Chinese Medicine, Second Military Medical University, Shanghai 200433, China; 2 Department of Rehabilitation Medicine, Changhai Hospital of Traditional Chinese Medicine, Second Military Medical University, Shanghai 200433, China

**Keywords:** miR-181a, Bufalin, Apoptosis, Prostate cancer, Traditional Chinese medicine

## Abstract

**Background:**

Bufalin is a major active compound of cinobufacini, which comes from dried toad venom and has been used for treatments of various cancers in China for many years. A number of studies have demonstrated that bufalin can induce apoptosis in some cancers. However, effects and mechanism of bufalin on prostate cancer cells remain unknown.

**Methods:**

Apoptosis assay was measured by the annexin-V/PI flow cytometric assay. Western blot was used to measure Caspase-3 and Bcl-2. qRT-PCR was used to measure the relative expression of miR-181a.

**Results:**

Bufalin was found to induce the expression of miR-181a, a small non-coding RNA believed to induce apoptosis by repressing its target gene, *BCL-2*. In prostate cancer PC-3cell line, bufalin-induced apoptosis can be largely attenuated by a miR-181a inhibitor, which blocked bufalin-induced Bcl-2 reduction and caspase-3 activation.

**Conclusions:**

Our dataindicatedthat miR-181a mediates bufalin-induced apoptosis in PC-3 cells. Thus, we presented here a new pharmacological mechanism for bufalin in anti-tumor therapy.

## Background

Cinobufacini is extracted from the skins and parotid venom glands of the toad Bufo bufo gargarizans *cantor* and has been widely used in clinical therapy for various cancers in China. The major pharmacologic constituents of cinobufacini are bufadienolides (which primarily include bufalin, cinobufagin, resibufogenin, bufotalin and lumichrome), alkaloids, biogenic amines, peptides and proteins [1]. Studies have suggested that some of its active compounds (e.g., bufalin and cinobufagin) exhibit significant antitumor activity, including inhibition of cell proliferation, induction of cell differentiation, induction of apoptosis, disruption of the cell cycle, inhibition of cancer angiogenesis, reversal of multi-drug resistance, and regulation of the immune response [2]. The mechanism of bufalin-induced apoptosis has been well investigated in various cancer cells. For example, bufalin was shown to induce apoptosis of human gastric cancer cells by inhibiting the PI3K/Akt signaling pathway [3]. In prostate cancer cells, bufalin significantly induces apoptosis through the p53- and Fas-mediated apoptotic pathways [4]. Bufalin was shown to induce ROS-mediated Bax translocation, mitochondrial permeability transition, and caspase-3 activation in human lung adenocarcinoma cells [5]. In an orthotopic transplantation tumor model of human hepatocellular carcinoma, bufalin showed significant anticancer action by regulating expression of apoptosis-related proteins, Bcl-2 and Bax [6]. Similarly, Takai et al. showed that bufalin-induced apoptosis was associated with levels of Bcl-2, Bcl-XL and caspase-9 in human endometrial and ovarian cancer cells [7].

MicroRNAs (miRNAs) are small, endogenous non-coding RNA molecules of ~ 22 nucleotides (nt) in length that can regulate gene expression. MiRNAs recognize and repress target mRNAs based on sequence complementarity, and are critical in regulating a variety of biological processes, including cell cycle, differentiation, development, and metabolism, as well as such diseases as diabetes, immuno- or neurodegenerative disorders, and cancer [8]. In cancer, miRNAs function as regulatory molecules, acting as oncogenes or tumor suppressors. Dysregulation of these miRNAs contributes to tumorigenesis by stimulating proliferation, angiogenesis and invasion [9-11].

MiR-181 was first identified in promoting B-cell differentiation when expressed in hematopoietic stem/progenitor cells [12]. Subsequently, the miR-181 family (miR-181a and miR-181b) was shown to function as tumor suppressors that triggered growth inhibition, induced apoptosis and inhibited invasion in glioma cells [13]. Ouyang et al. showed miR-181 to induce apoptosis by targeting multiple Bcl-2 family members in astrocytes [14]. Recently, several studies further showed that by targeting various multiple anti-apoptosisgenes, such as *BCL-2*, miR-181 significantly enhances drug- or radiation-induced apoptosis in various cancer cells [15-20]. In chronic myeloid leukemia (CML), the *RalA* gene was reported as a direct target of miR-181a, and is associated with cell proliferation, G_2_-phase arrest and apoptosis [21].

Here, we report that bufalin treatment could induce miR-181a expression. We also show that miR-181a contributes to bufalin-induced apoptosis in prostate cancer cells. Thus, our study illustrated a new pharmacological mechanism for bufalin in anti-tumor therapy.

## Methods

### Cell culture and treatment

Human prostate carcinoma PC-3 cells were maintained in Ham’s F-12 medium (Invitrogen, Carlsbad, CA, USA) supplemented with 10% fetal bovine serum (Invitrogen, Carlsbad, CA, USA). *Bufalin* (Sigma-Aldrich, St. Louis, MO, USA) was dissolved in *DMSO* and stocked in 1 mM solution. Cells with 80–*90*% *confluence in* 12-well plates were treated with indicated concentrations of bufalinfor 24 hours. When combined with miR-181a inhibitor, 50 or 100 μM of miR-181a inhibitor was transfectedinto cells (~70% *confluence in* 12-well plates*)* 12 hours before bufalin treatment. MiR-181a, miR-NC and their inhibitors were purchased from GenePharma (GenePharma, Shanghai, China). Sequence of miR-NC was from *C. elegans*and has no known similar sequence in the human genome. Transfection was performed using Lipofectamine™ RNAiMAX (Invitrogen, Carlsbad, CA, USA).

### RNA isolation and quantitative real-time PCR

Total RNA was isolated by Trizolreagent (Invitrogen, Carlsbad, CA, USA) according to the user’s guide specifically for short RNAs. Briefly, cells were homogenized by *RNApro* reagent. After phase separation by chloroform, 2.5 volume of alcohol was added to the aqueous phase to precipitate total RNA containing short RNA. Total RNA was then recovered by centrifuge and dissolved in nuclease-free water. Two micrograms of total RNA was tailed and reverse transcribed by NCode™ EXPRESS SYBR® GreenER™ miRNA qRT-PCR Kit (Invitrogen, Carlsbad, CA, USA) according to the user’s manual. Quantitative real-time PCR was performed by miRNA specific primers (Additional file 1: Table S1). All Ct values of miRNAs were normalized to 18S rRNA. The 2^−ΔΔCt^ method was used to calculate relative expression level of miRNAs.

### Apoptosis assay

The apoptosis assay was performed with an annexin-V-FITC apoptosis detection kit (Sigma-Aldrich, St. Louis, MO, USA) according to the user’s manual. Cells after different time treatments were washed by twice with PBS (Phosphate Buffered Saline) buffer. Cells were then resuspended in 1 × binding buffer at a concentration of ~1 × 10^6^ cells/ml, and 5 μl of Annexin V FITC conjugate and 10 μl of propidium iodide (PI) solution were added to each 500-μl cell suspension. Cells were stained by Annexin-V-FITC/PI for 10 min at room temperature. Stained samples were analyzed using MoFlo XDP flow cytometer (Beckman Coulter, Brea, CA, USA) and the apoptosis rate was determined using Flowjo software (Tree Star, Ashland, OR, USA).

### Western blotting

Cells were washed with PBS and lysed in RIPA buffer. Cell lysate aliquots (10 μg) were separated on a 10% SDS-PAGE gel and transferred to PVDF membrane. Primary antibodies for Bcl-2, Caspase-3, RalA and β-actin were purchased from Abcam (Abcam, Cambridge, MA, USA). Secondary antibody coupled with HRP was from Sigma (Sigma-Aldrich, St. Louis, MO, USA). Membrane was visualized by ECL PicoLightChemiluminescence kit (Promoton, Shanghai, China). Membrane was then exposed to X-ray film in dark room.

### Caspase-3 activity assay

Caspase-3 activity assay was performed by Caspase-Glo® 3/7 Assay kit (Promega, Madison, WI, USA) in 96-well plate according to the user’s manual. Luminescence was measured on a Mithras Multimode Microplate Reader LB 940 (Berthold, Calmbacher, Germany).

## Results

### Bufalin induced the expression of miR-181a

**Figure 1:**
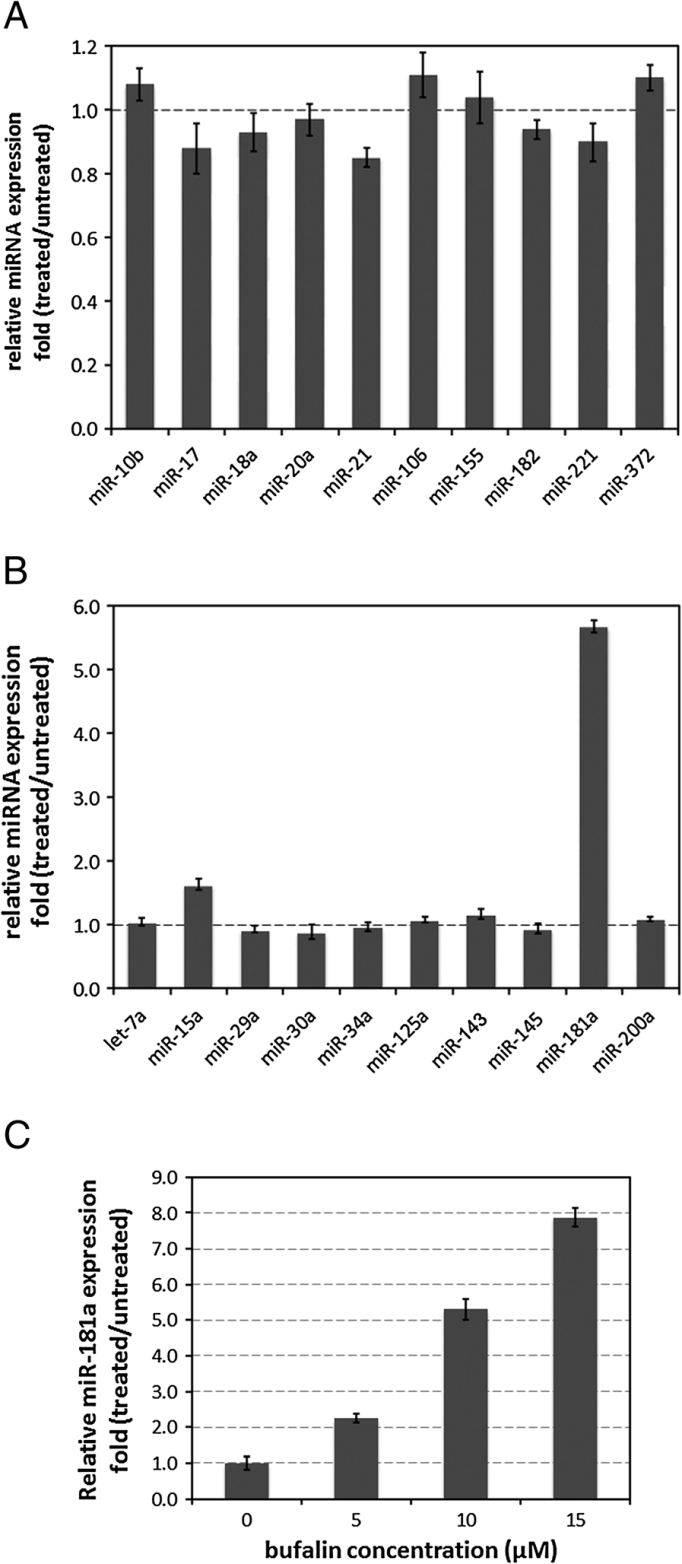
**MiR-181a was induced by bufalin in PC-3 cells. A**. Expression of 10 oncogenic miRNAs in PC-3 cellstreated withbufalin at a concentration of 15 μM for 24 hours. **B**. Expression of 10 tumor-suppressor miRNAs in PC-3 cells treated with bufalin at 15 μM for 24 hours. **C**. Expression of miR-181a in PC-3 cells treated with bufalinat 0, 5, 10, and 15 μM for 24 hours. Vertical axes indicate fold-changes in expression of miRNAs in cells, with and without bufalin treatment. All data were from three replications; error bar indicatedstandard error of each measurement.

To test if certain miRNAs are involved in bufalin-induced anti-tumor activity, two sets of cancer-related miRNAs—oncogenes (Figure 1A), and so-called tumor suppressors (Figure 1B)—were screened by quantitative real-time PCR in PC-3 cells after bufalin treatment, at aconcentration of 10 μM [9-11,22]. Bufalin showed no significant effects on 10 screened oncogenicmiRNAs (Figure 1A). In the second set of miRNAs, which usually act as tumor suppressors, expression level of two miRNAs increased after bufalin treatment (Figure 1B). MiR-181a increased more than fivefolds compared to its basal expressionlevel, whereas miR-15a only increased by ~50%. We focused on miR-181a because it is the most significant induced miRNA in our study. We further determined miR-181a levels to be induced at different bufalin concentrations. MiR-181a expression was significantly induced by bufalin in a dose-dependent manner (Figure 1C). The miR-181a level was induced to nearly eight foldsas its basal level after treatment by bufalin at a concentration of 15 μM.

### MiR-181a inhibitor attenuated bufalin-induced apoptosis

**Figure 2:**
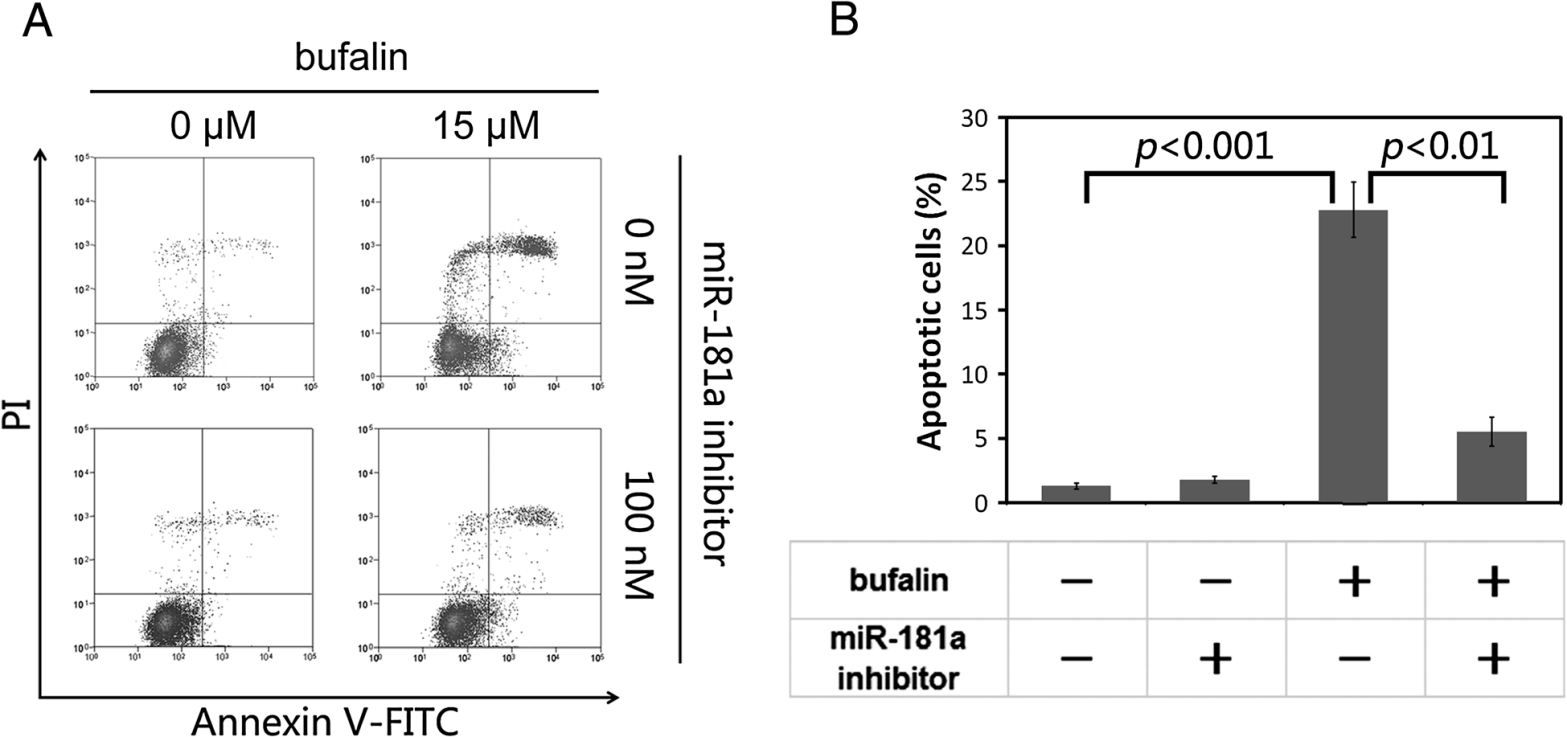
**MiR-181a inhibitor attenuatedbufalin-induced apoptosis in PC-3 cell. A**. Apoptotic cells were stained by Annexin-V-FITC/PI and assayed by flow cytometer. Bufalin induced significant apoptosis that was effectively attenuated by miR-181a inhibitor. **B**. Statistical histogram from A was shown. *P* values were calculated byStudent’s *t*-test, based on three replications.

Both bufalin and miR-181a could induce apoptosis in various cancer cells [4-7,14-21,23-26]. As bufalin can induce miR-181a expression, we speculated that bufalin-induced apoptosis may be mediated, at least partly, by miR-181a. To address this point, we tried to use miR-181a inhibitor to block bufalin-induced apoptosis. Bufalin treatment resulted in a 22.8% apoptosis rate in PC-3 cells, whereas the apoptosis rate decreased to 5.5% in cells transfected with miR-181a inhibitor (Figure 2). These data indicated that inhibition of miR-181a activity could attenuate bufalin-induced apoptosis in PC-3 cells.

### MiR-181a inhibitor can reverse bufalin-induced Bcl-2 decrease

**Figure 3:**
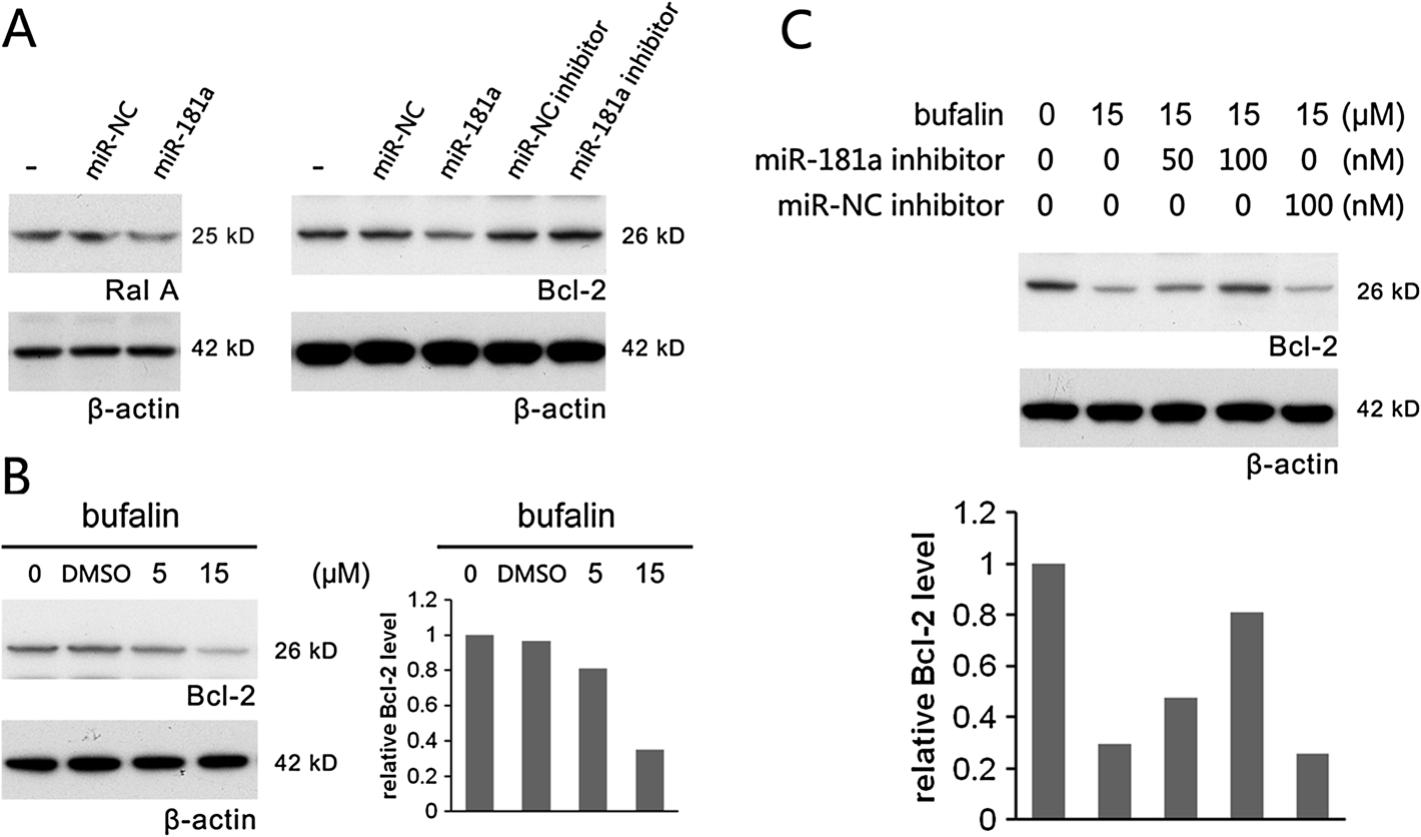
**MiR-181a inhibitor blocked bufalin-induced Bcl-2 reduction in PC-3 cell. A**. MiR-181a could repress the expression of *Bcl-2* and *RalA*. **B**. Bufalin reduced Bcl-2 protein in a dose-dependent manner. The relative level of Bcl-2 protein in the western blot was normalized to β-actin and was shown in right panel. **C**. Bufalin-induced Bcl-2 reduction was largely blocked by miR-181a inhibitor transfection. The relative level of Bcl-2 protein in the western blot was normalized to β-actin and was shown in lower panel. β-actin served as internal control; miR-NC indicated a negative miRNA control.

MiR-181a was believed to induce apoptosis by repressing its target genes, *Bcl-2*and *RalA*[14-21]. Transfection of miR-181a was shown to significantly down-regulate Bcl-2 and RalA protein (Figure 3A). Similarly, bufalin treatment decreasedBcl-2 proteinin a dose-dependent manner (Figure 3B); at 15 μM of bufalin, Bcl-2 protein level reduced by about 70%. Furthermore, miR-181a inhibitor can reverse bufalin-induced Bcl-2 reduction (Figure 3C). Transfection with 100 μMof miR-181a inhibitor raised Bcl-2 protein to ~80% of normal level. Anegative control inhibitor showed no reverse effect on Bcl-2 protein level after bufalin treatment. These results indicated that induced miR-181a mediates downstream bufalin-induced apoptosis by repressing Bcl-2 protein in PC-3 cells.

### MiR-181a inhibitor can reduce bufalin-induced caspase-3 activity

**Figure 4:**
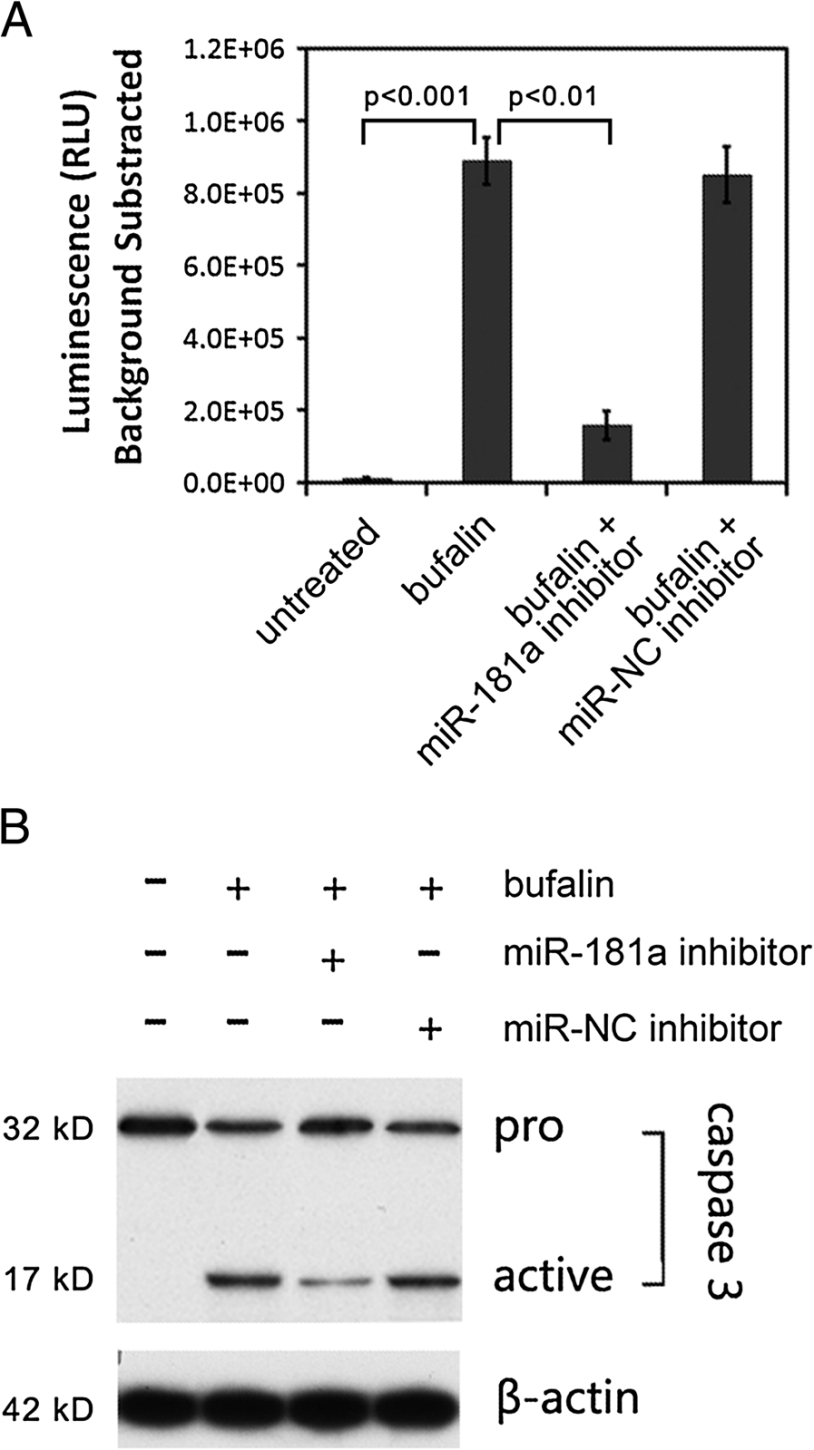
**Bufalin-induced caspase-3 activity was reduced by miR-181a inhibitor transfection in PC-3 cell. A**. Caspase-3 activity was assayed by a caspase-3 substrate cleavage based luminescence kit. Histogram showed the background subtracted luminescence value. *P* values were calculated byStudent’s *t*-test, based on three replications. **B**. Western blot showed that the active form of caspase-3 was reduced by miR-181a inhibitor transfection. β-actin served as internal control; miR-NC indicated a negative miRNA control.

Bcl-2 is an anti-apoptosis protein and its decrease usually triggers mitochondrion mediated apoptosis pathway by caspase-3 proteins activation. Therefore, we also assayed caspase-3 activity by cleavage of aminoluciferin-coupled caspase-3 substrate in lysate of PC-3 cells treated with or without bufalin. Upon caspase-3 activation, aminoluciferin-coupled caspase-3 substrate was cleaved and aminoluciferin, a substrate of luciferase, is released, resulting in the luciferase reaction and production of luminescence. After bufalin treatment, miR-181a inhibitor transfected cell lysate, showed only ~20% caspase-3 activity when compared with untransfected lysate; whereas transfection with a negative control inhibitor did not reduce bufalin-induced caspase-3 activity (Figure 4A). The cell lysates were further subjected to western blot analysis with a caspase-3 antibody that recognizes both pro- and cleaved caspase-3. After bufalin treatment, pro-caspase-3 was cleaved to a smaller active form that can lead to apoptosis. MiR-181a inhibitor, however, largely reduced the activating cleavage of pro-caspase-3 and the level of the active form of caspase-3 (Figure 4B).

## Discussion

Cinobufacini, is a form of traditional Chinese medicine, and has been approved by the Chinese State Food and Drug Administration (SFDA) for many years. Cinobufacini injection is widely used in China to treat patients with various cancers [27]. Many clinical trials have shown it to effectively shrink lesions and improve patients’ survival rate and quality of life. Bufalin, as a major active compound of cinobufacini, was considered to have great effect on tumors, including inhibition of proliferation and cancer angiogenesis, induction of differentiation and apoptosis, disruption of cell cycle, reversal of multi-drug resistance, and regulation of immune response [6]. Although various studies present the mechanism by which bufalin induces apoptosis in cancer cells, the anti-tumor activity of bufalin and miRNAs in inducing miR-181a expression had not been shown before this study.

Many miRNAs regulate various processes in tumorigenesis, including apoptosis and metastasis, and have received increasing attention in cancer research. To test if miRNA pathways crosstalk with the pharmacologic action of bufalin in cancers, we screened expression of some cancer-related miRNAs in PC-3 cells after bufalin treatment, and observed miR-181a expression to significantly increase in a dose-dependent manner. We also showed miR-181 to induce significant apoptosis through down-regulation of Bcl-2 protein. Furthermore, miR-181a inhibitor largely attenuated bufalin-induced apoptosis. Our results indicate that miR-181a mediates a downstream, bufalin-induced apoptosis pathway, and suggest a more detailed model for bufalin-induced apoptosis in which bufalin induces expression of miR-181a, which in turn inhibits Bcl-2 protein, resulting in apoptosis.

## Conclusions

Based on our result, we presented here a more detailed model for bufalin-induced apoptosis. Bufalin treatment induced the expression of miR-181a, which in turn inhibited Bcl-2 protein and resulted in cell apoptosis.

## Competing interests

The authors have no actual or potential conflict of interest associated with this work.

## Authors’ contributions

XFZ, FFF and ZC participated in the design of the study data analyses and manuscript preparation. QL, YBM, YYG and FFF conducted the assays and analyses. All authors read and approved the final manuscript.

## Pre-publication history

The pre-publication history for this paper can be accessed here:

http://www.biomedcentral.com/1472-6882/13/325/prepub

## Supplementary Material

Additional file 1: Table S1 Primers used in this study. Click here for file
