# Supplementary material for: MiR-181a contributes to bufalin-induced apoptosis in PC-3 prostate cancer cells
Source: BMC Complement Altern Med. 2013 Nov 23;13:325. doi: 10.1186/1472-6882-13-325 (PMC4222721; doi:10.1186/1472-6882-13-325)
Supplement: Additional file 1: Table S1 — Primers used in this study. [file 1472-6882-13-325-S1.doc]

Table S1. Primers used in this study.

| miRNA name | sequence (5-3) |
| --- | --- |
| miR-10b | ACCCTGTAGAACCGAATTTGT |
| miR-17 | CAAAGTGCTTACAGTGCAGGT |
| miR-18a | TAAGGTGCATCTAGTGCAGAT |
| miR-20a | AAGTGCTTATAGTGCAGGTAG |
| miR-21 | AGCTTATCAGACTGATGTTGA |
| miR-106 | AAGTGCTTACAGTGCAGGTAG |
| miR-155 | TAATGCTAATCGTGATAGGGG |
| miR-182 | TTTGGCAATGGTAGAACTCACA |
| miR-221 | GCTACATTGTCTGCTGGGTTTC |
| miR-372 | AGTGCTGCGACATTTGAGCGT |
| let-7a | TGAGGTAGTAGGTTGTATAGT |
| miR-15a | AGCAGCACATAATGGTTTGTG |
| miR-29a | TAGCACCATCTGAAATCGGTT |
| miR-30a | GTAAACATCCTCGACTGGAAG |
| miR-34a | GGCAGTGTCTTAGCTGGTTGT |
| miR-125a | CCTGAGACCCTTTAACCTGT |
| miR-143 | TGAGATGAAGCACTGTAGCTC |
| miR-145 | TCCAGTTTTCCCAGGAATCCCT |
| miR-181a | ACATTCAACGCTGTCGGTGAG |
| miR-200a | TAACACTGTCTGGTAACGATG |
| 18S rRNA | GTGAACCTGCGGAAGGATCA |
